# Prevention and Mitigation of Rural Higher Education Dropout in Colombia: A Dynamic Performance Management Approach.

**DOI:** 10.12688/f1000research.132267.2

**Published:** 2023-06-01

**Authors:** Alfredo Guzman Rincón, Sandra Barragán, Federico Cosenz, Favio Cala Vitery

**Affiliations:** 1 Corporacion Universitaria de Asturias, Bogota, Bogota, Colombia; 2 Universidad de Bogota Jorge Tadeo Lozano, Bogota, Bogota, Colombia; 3 Universita degli Studi di Palermo, Palermo, Sicily, Italy

**Keywords:** Simulation models, higher education, rural areas, Dynamic Performance Management, dropping out

## Abstract

**Background: **Dropout in higher education is a socio-educational phenomenon that has the scope to limit the benefits of education as well as to widen social disparities. For this reason, governments have implemented various public policies for its prevention and mitigation. However, in rural populations, such policies have proven to be ineffective. The aim of this paper is to simulate public policy scenarios for the treatment of school dropout in rural higher education in Colombia from a Dynamic Performance Management approach.

**Methodology: **To achieve the aim, a parameterised simulation model was designed with data from Colombian state entities in rural higher education. Five simulations were carried out. The analysis of the results was carried out using descriptive statistics and comparison of means using the Wilcoxon Sign Rank statistic.

**Results: **The adoption of such an approach based on simulations suggests that policies to expand the coverage of educational credits and financial support, as well as the addition of a family income subsidy, allow for a reduction in the number of dropouts.

**Conclusions: **A dynamic, data-driven approach can be effective in preventing and mitigating dropout in these areas. It also highlights the importance of identifying the key factors contributing to dropout. The results also suggest that government policies can have a significant impact on school retention in rural areas.

## Introduction

Apart from a few success stories of personalities such as Steve Jobs, Mark Zuckerberg, Larry Ellison, and Bill Gates, who did not complete higher education, ^1^ student dropout has been a consistent challenge for policy decision makers globally. ^2^^,^^3^ Dropping out of higher education can limit the benefits that come with educational level for society and exacerbate social disparities in certain areas. ^2^^,^^4^^,^^5^ The negative effects of dropout in higher education are related to learning factors, which can make it impossible to obtain better jobs in the long run due to a lack of educational capital. ^6^^,^^7^ This makes it difficult to improve the average income level of the population, ^8^ decreases the skilled workforce, ^9^ and destroys social capital, ^10^ among other things. Dropout in higher education also hinders efforts to reinforce civic and democratic values. ^10^ Moreover, dropout from higher education leads to an increase in poverty rates as students and their families have to bear the sunk costs related to education. ^5^

Based on the literature, certain student populations experience dropout in higher education more frequently, due to the interaction of individual, socioeconomic, academic, and institutional variables that induce them to abandon their education prematurely, as in the case of rural populations. ^5^^,^^11^^–^^14^ In response, both developed and developing countries have sought to mitigate and prevent dropout in rural student populations. ^5^ Western states have focused on designing, developing, and implementing public policies that fund tuition and other educational expenses, as well as creating quality standards that evaluate the role of Higher Education Institutions (HEIs) in mitigating and preventing dropout. ^2^^,^^15^

Despite the existence of public policies aimed at reducing the dropout rate in rural higher education, the rate remains high, averaging over 50% in countries such as Colombia, India, and South Africa. ^5^^,^^11^^,^^16^^–^^18^ The root causes of this phenomenon are often linked to the ineffective implementation of existing policies, or the absence of comprehensive policies that address critical individual, socioeconomic, academic, and institutional variables (or combinations of them) that lead to students dropping out.

In order to address this issue, it is necessary to evaluate the treatment of other explanatory variables that can help to combat dropout in higher education within the context of public policy. However, literature linking dropout to rural populations and public policies is scarce, so the academic community has provided feedback to decision-makers aimed at identifying variables that explain the possible causes of rural higher education student dropout. ^2^^,^^13^^,^^19^^–^^22^ An example of this was the study carried out by Guzmán *et al*., ^2^^,^^5^ in which a model based on systems thinking was used to indicate to public policy decision-makers, the need to improve student performance at previous academic levels, improve connectivity and access to information and communication technologies, and generate financial support, among others. Nonetheless, it is still unclear how incorporating these variables into public policies could positively impact the prevention and mitigation of dropout in the rural student population. This is due to the fact that most related studies of dropout in rural higher education make use of cross-sectional methods (e.g., Refs. 2, 12, 13, 23), and those that are longitudinal tend to be ex post facto non-predictive in nature, as in the study by Faizullina *et al*., ^24^ where data was taken from 2,388 students analysing their retention and dropout status, in order to find out the causes of dropout. Thus, there is a need for models that provide information over time to public policy makers on the long-term impact of their decisions in addressing dropout in rural higher education.

Against this backdrop, the objective of this article is to simulate public policy scenarios for the treatment of school dropout in rural higher education in Colombia from a Dynamic Performance Management (DPM) approach. Given the complexity of the global educational system, the study is limited to Colombia due to the high dropout rate in its rural areas, which can be as high as 50% per year and in some departments (states or provinces) can be even higher than 80%. ^18^^,^^25^ This study aims to provide new insights into this educational issue, which is heavily influenced by social disparities resulting from armed conflicts, drug trafficking, and State abandonment, among other factors. ^2^^,^^5^ The study seeks to provide possible solutions for preventing and mitigating dropout in the rural student population by designing a model that evaluates the impact of public policy decisions prior to their implementation. Furthermore, it aims to contribute to the field of study by using DPM modelling to understand how the treatment of explanatory variables for dropout affects students over time.

As the study focuses specifically on rural higher education in Colombia, the model is limited to the explanatory variables of dropout that the Ministry of National Education evaluates over time. Therefore, subsequent sections will not consider all variables studied within the framework of this educational phenomenon. This article is organized into four sections. The first section presents the theoretical framework and Colombian public policies for the treatment and mitigation of dropout. The second section describes the methodology used and the scenarios to be simulated. The third section outlines the results including the DPM Chart, Causal Loop Diagram, Forrester Diagram, Mathematical Model, and the results of the simulations. Finally, the fourth section provides a discussion and conclusions.

## Theoretical framework

### Dropout in rural higher education

Dropout is a complex and multi-causal phenomenon that involves various actors within the educational system, including State entities, HEIs, students, their families, and financial institutions. There are numerous variables that explain this phenomenon, such as academic performance, socioeconomic status, age, gender, attendance at wellness programs, etc., which can influence students’ decision to terminate their training process prematurely in higher education. ^2^ Due to the interdisciplinary nature of the study of dropout, there are multiple conceptualizations of this phenomenon, depending on the actor analysing it.

Academic conceptualizations provide an overall epistemic value. Among these definitions, the Alfa Guía Project stands out, which sought to contribute to the reduction of dropout rates in Higher Education through the cooperative work of a Network of Educational Institutions formed by 138 different institutions from 30 different countries. Thus, dropout was conceptualised from this project where dropout is defined as

“the cessation of the relationship between the student and the training program that leads to a degree of higher education before it is achieved. A complex, multidimensional, and systemic event can be understood as cause or effect, failure or reorientation of a training process, mandatory choice or response, or as an indicator of the quality of the educational system”. ^26^

In contrast, various countries such as Colombia, Spain, United Kingdom and United States, use technical definitions to facilitate the counting of dropout students and identify variables that explain the phenomenon and its causes. In Colombia, for example, dropout is defined based on the number of academic periods that a student was not linked to an HEI. A student is considered to have dropped out if they did not enrol in the program in two consecutive periods and did not graduate or withdrew for disciplinary reasons. ^27^

Both the academic community and the Colombian definition are not exclusive but complement each other to analyse dropout in higher education from different perspectives. To analyse dropout, public policy development and academic research have used four determinants to conglomerate the explanatory variables of this phenomenon: individual, socioeconomic, academic, and institutional. ^2^^,^^5^^,^^28^^–^^31^ These determinants are defined as follows by Guzman *et al.* ^2^:

(1) Individual: It corresponds to the characteristics of the students and their personal environment, which directly and indirectly affect dropout.
(2) Socioeconomic: It refers to the influence of the social and economic context in which the students develop and that can lead them to not complete their training.
(3) Academic: It conglomerates the variables related to learning outcomes, competencies, abilities, performance, and other aspects of the teaching and learning process at all levels of education completed or in progress, both formal and informal.
(4) Institutional: It concerns the aspects and institutional policies of HEIs that can lead the students to finish their training early.

In the context of global dropout rates, research on students in rural higher education has shown that women are typically responsible for domestic and childcare duties, leading to less time for studying, while men’s study time is reduced due to work obligations. ^2^^,^^21^^,^^32^ Additionally, entering higher education at a later age in rural areas often means increased family and work responsibilities, which can result in dropping out of school. ^32^^,^^33^ A study by Guzmán *et al.,* ^2^ found that in Colombia the lack of support structures due to low social capital in rural families was associated with students’ intentions to drop out, a finding also reported by De Hart and Venter in South Africa. ^32^ Low social capital is connected to parents’ educational levels and, subsequently, to the delayed entry into higher education, which is attributed to lower perceived value of higher education in rural societies. ^32^ Due to social disparities, rural students who drop out, as well as their families, generally have lower incomes than the national average, for the case of the United States, ^12^ and sometimes even below the minimum wage. ^5^ Such factors make it challenging to afford tuition fees, educational expenses, and overall training development in a fair and equitable manner compared to students’ urban counterparts. ^34^

Inequality is not limited solely to socioeconomic factors but also affects academic processes themselves. Rural students often have lower academic performance at earlier levels, inadequate teacher training, and limited access to education-related services and technology. ^23^ To address this issue, studies suggest that implementing Permanence and Timely Graduation Plans (P&GO according to its Spanish acronym) in HEIs can help prevent and reduce dropout rates among rural students. ^35^ Other factors related to rural student dropout include dissatisfaction with the chosen academic program, ^33^ inadequate information about the program prior to enrolment, ^21^ limited access to resources and technology at HEIs, ^36^ and the type of school from which they graduated. ^37^

It is recognised that only some of the variables studied are linked to the development of public and institutional policies because statistical information is needed for their treatment or simply because of the level of complexity that makes direct state intervention difficult. Examples of this type of variable are the level of self-regulation, student motivation, satisfaction with their studies, etc. Consequently, the model proposed in the following sections focuses on those variables for which statistical information from state information systems is available and which can be intervened through public policies.

### Colombian public policies for the treatment and mitigation of dropout in higher education and rural areas

When discussing public policies aimed at preventing and mitigating student dropout in higher education, it is important to revisit the initial efforts made by the Colombian Ministry of National Education in 2003 to understand and contextualize them. That year, the State set out to:

(1) Facilitate student migration between programs.
(2) Improve regulatory mechanisms for admitting students who are simultaneously studying at other HEIs.
(3) Increase and enhance the information provided to applicants about the programs offered.
(4) Create financial aid programs for low-income students from other cities.
(5) Promote prior vocational or professional guidance. ^38^

To this end, the Ministry of National Education developed the Higher Education Dropout Prevention System (SPADIES, according to its Spanish acronym) in the first phase to determine the actual dropout rate and the possible variables that influenced this phenomenon. The State then designed a public policy in which it sought to finance higher education through credits, ^5^ in order to mitigate socioeconomic variables related to dropout. In addition, as the State regulates the educational system, it established a series of policies for HEIs related to educational quality regarding dropout rates, access, permanence, and timely graduation. HEIs created Early Warning Systems (SAT, according to its Spanish acronym) and P&GO to accurately identify students with the intention of dropping out and intervene timely. ^18^^,^^39^

As a result of the implementation of these policies, the dropout rate in Colombia ranged from 7.56% to 12.8% for the period spanning 2017 to 2021, ^40^ which is relatively low compared to the situation in other Latin American countries or in the Organization for Economic Cooperation and Development (OECD), where dropout rates of 54% and 64.5%, respectively, were registered. ^41^^,^^42^ However, when the analysis is focused on rural areas, the reality is different. It has been estimated that the dropout rate for training programs aimed at rural students reaches 50% at the national level. ^25^ Nevertheless, the dropout rate varies between departments, with the poorest ones experiencing the most significant challenges. Examples are the departments of Putumayo, La Guajira and Arauca, where the dropout rate in higher education was 80.2% and 55.6%, respectively. ^25^

At present, there are no differentiated state public policies for the rural population. Hence, it is necessary to analyse through modelling which variables should be addressed to allow for students’ persistence and timely graduation in rural areas.

## Methods

### Design

To achieve the objective of this article, we utilized the DPM approach, which combines planning and control systems with System Dynamics modelling. ^43^^,^^44^ The DPM is a management approach that focuses on adaptability and continuous improvement. It is characterized by not relying solely on static measurements and predefined objectives but recognizes the dynamism of the elements that make up the system. The DPM approach distinguishes itself from traditional System Dynamics modeling methods (Causal Loop Diagram and Forrester Diagram) because it is based on the optimization of strategic resources. ^45^^–^^47^ This allows for not only recognizing causal relationships but also actively adapting and improving the performance of the system in response to changes in the environment. ^48^ Additionally, the DPM approach identifies the outputs and outcomes of the system, which is crucial for establishing relevant performance drivers aligned with strategic resources. ^47^ Finally, by utilizing strategic resources, performance drivers, and final outcomes, the DPM approach expands the boundaries of the system for a better understanding of the relationship between the elements of the system under study and other directly and indirectly interacting systems. ^48^ The conceptualization of the central elements of the DPM approach is presented below.

(1) Strategic resources: These are active elements and capabilities of a system that are used to achieve objectives represented by final outcomes. These resources can be tangible or intangible. Tangible resources include financial assets, technology, infrastructure, facilities, and equipment. Intangible resources encompass specialized knowledge, skills, customer and supplier relationships, intellectual property, and organizational culture.
(2) Performance drivers: These are the factors that mediate the system’s performance as they act as points of connection between strategic resources and final outcomes. These drivers can be internal or external and have a significant impact on the system’s ability to achieve its objectives.
(3) End results: These are the results generated by the system and have the ability to change the initial state of strategic resources. These outcomes are divided into two categories. The first category is outputs, which are results generated directly by the system and depend solely on it. The second category is outcomes, which are results influenced by the system but not solely dependent on it. An example of an outcome category is the quality of life, which is affected by multiple systems such as education, health, public safety, infrastructure, among others.

The DPM approach involves identifying auxiliary variables, flows, and levels within a system or subsystems, as well as the relationships between these elements, to recreate their structures. Equations are then developed to simulate possible system behaviours over time. ^44^ In this case, we used this approach to model the social and higher education structures and measure their performance based on the number of enrolled, graduated, and dropout rural students.

Building on previous works such as Bivona and Cosenz, ^49^ Herrera *et al.,* ^50^^,^^51^ Bivona, ^52^ Cosenz *et al.,* ^53^ Bianchi *et al.,* ^54^ Feng *et al.,* ^55^ and Yin *et al.,* ^56^ we developed a model in five phases. ^57^ The first phase involved identifying the strategic resources, performance drivers, and results and documenting the DPM Chart. The second phase focused on developing the Causal Loop Diagram to establish the behaviour of the system and interactions between variables based on the DPM Chart. In the third phase, we created the Forrester or Stocks and Flows Diagram to synthesize the differential equations and establish the system’s structure, achieving dynamic behaviour as a function of time. The fourth phase involved defining equations that replicate the system’s behaviour, with software-generated equations chosen for consistency and replicability (for all equations, see *Extended data* ^68^). The fifth and final phase tested the validation of the model.

This validation was performed through sensitivity analysis, which is a validation method that “evaluates the changes in the model’s behavior when systematically varying the input parameters”. ^58^ This validation test reveals the parameters to which the model is highly sensitive. Thus, a model is considered valid when the numerical values of the variables under study change, but the model’s behavior remains consistent. This validation test can reveal the degree of robustness in the model’s behavior and, therefore, indicate the extent to which recommendations based on the model could be affected by uncertainty in parameter values. For the purposes of this study, the following variables were modified by ±10% from their initial parameter values (see Table 1): high school graduation rate, higher education entry rate, graduation rate, coverage of educational credits and related grants, and P&GO coverage. If the modification in a variable resulted in negative values, the minimum value for sensitivity analysis was set to 0. A total of 50 scenarios were simulated with a uniform distribution for all variables. This information is graphically presented in the results section.

**Table 1.** Data and sources of model parameterization.

Variable	Type	Units	Value	Source
Average age of entry or average age of admission	Stock	Years	25	Ministry of National Education ^40^
Impact rate of average age on family obligations	Auxiliar variable	Hours	GRAPH (Average_age_of_entry) (17.00, 6.00), (18.00, 7.00), (19.00, 7.00), (20.00, 7.00), (21.00, 8.00), (22.00, 8.00), (23.00, 8.00), (24.00, 9.00), (25.00, 9.00), (26.00, 9.00), (27.00, 10.00), (28.00, 10.00), (29.00, 10.00), (30.00, 11.00), (31.00, 11.00), (32.00, 12.00), (33.00, 12.00), (34.00, 12.00), (35.00, 13.00), (36.00, 13.00), (37.00, 14.00), (38.00, 14.00), (39.00, 14.00), (40.00, 14.00), (41.00, 15.00), (42.00, 15.00), (43.00, 15.00), (44.00, 15.00), (45.00, 15.00), (46.00, 15.00), (47.00, 15.00), (48.00, 15.00), (49.00, 14.00), (50.00, 14.00), (51.00, 14.00), (52.00, 14.00), (53.00, 13.00), (54.00, 13.00), (55.00, 12.00), (56.00, 12.00), (57.00, 12.00), (58.00, 11.00), (59.00, 11.00), (60.00, 10.00), (61.00, 10.00), (62.00, 10.00), (63.00, 9.00), (64.00, 9.00), (65.00, 8.00), (66.00, 8.00), (67.00, 8.00), (68.00, 7.00), (69.00, 7.00), (70.00, 7.00), (71.00, 7.00), (72.00, 6.00), (73.00, 6.00), (74.00, 6.00)	Guzmán *et al.* ^59^
Average time spent on family obligations	Stock	Hours	9	Ministry of National Education ^40^
Impact rate of average household income on labour obligations	Auxiliar variable	Hours	GRAPH (Household_income_average) (0, 48.00), (300000, 48.00), (600000, 48.00), (900000, 48.00), (1200000, 48.00), (1500000, 48.00), (1800000, 21.45), (2100000, 18.71), (2400000, 15.67), (2700000, 13.32), (3000000, 13.32)	Guzmán *et al.* ^5^
Average time of labour obligations	Stock	Hours	40	Ministry of National Education ^40^
Impact rate of average age on work obligations	Auxiliar variable	Hours	GRAPH (Average_age_of_entry) (17.00, 6.00), (18.00, 7.00), (19.00, 7.00), (20.00, 7.00), (21.00, 8.00), (22.00, 8.00), (23.00, 8.00), (24.00, 9.00), (25.00, 9.00), (26.00, 9.00), (27.00, 10.00), (28.00, 10.00), (29.00, 10.00), (30.00, 11.00), (31.00, 11.00), (32.00, 12.00), (33.00, 12.00), (34.00, 12.00), (35.00, 13.00), (36.00, 13.00), (37.00, 14.00), (38.00, 14.00), (39.00, 14.00), (40.00, 14.00), (41.00, 15.00), (42.00, 15.00), (43.00, 15.00), (44.00, 15.00), (45.00, 15.00), (46.00, 15.00), (47.00, 15.00), (48.00, 15.00), (49.00, 14.00), (50.00, 14.00), (51.00, 14.00), (52.00, 14.00), (53.00, 13.00), (54.00, 13.00), (55.00, 12.00), (56.00, 12.00), (57.00, 12.00), (58.00, 11.00), (59.00, 11.00), (60.00, 10.00), (61.00, 10.00), (62.00, 10.00), (63.00, 9.00), (64.00, 9.00), (65.00, 8.00), (66.00, 8.00), (67.00, 8.00), (68.00, 7.00), (69.00, 7.00), (70.00, 7.00), (71.00, 7.00), (72.00, 6.00), (73.00, 6.00), (74.00, 6.00)	Guzmán *et al.* ^59^
Average time spent studying	Stock	Hours	35	Guzmán *et al.* ^59^
High school graduates	Stock	Students	108,587	Ministry of National Education ^25^
High school graduation rate	Auxiliar variable	%	2.5	Ministry of National Education ^25^
Students enrolled	Stock	Students	26,818	Ministry of National Education ^25^
Dropout rate by time spent studying	Auxiliar variable	%	GRAPH (Average_time_spent_studying) (0.0, 0.246), (18.0, 0.218), (36.0, 0.194), (54.0, 0.181), (72.0, 0.161), (90.0, 0.137), (108.0, 0.101), (126.0, 0.085), (144.0, 0.069), (162.0, 0.056), (180.0, 0.056)	Guzmán *et al.* ^59^
Drop-out rate due to ineffective P&GO interventions	Auxiliar variable	%	GRAPH(P&GO_Interventions) (0, 0.173), (1000000, 0.133), (2000000, 0.108), (3000000, 0.096), (4000000, 0.086), (5000000, 0.063), (6000000, 0.049), (7000000, 0.045), (8000000, 0.039), (9000000, 0.033), (10000000, 0.033)	Guzmán *et al.* ^59^
Dropout rate due to lack of credits and educational grants	Auxiliar variable	%	GRAPH (Educational_credits_and_grants) (0, 0.153), (20000, 0.109), (40000, 0.077), (60000, 0.052), (80000, 0.024), (100000, 0.012), (120000, 0.000), (140000, 0.000), (160000, 0.000), (180000, 0.000), (200000, 0.000)	Guzmán *et al.* ^59^
Dropout students	Stock	Students	13,409	Ministry of National Education ^40^
Graduates	Stock	Students	5,922	Ministry of National Education ^40^
Graduation rate	Auxiliar variable	%	4.5%	Ministry of National Education ^25^
Impact rate of graduates on household income	Auxiliar variable	%	GRAPH (Graduates) (0, 0.073), (200000, 0.077), (400000, 0.097), (600000, 0.121), (800000, 0.133), (1000000, 0.153), (1200000, 0.181), (1400000, 0.214), (1600000, 0.246), (1800000, 0.294), (2000000, 0.355)	Guzmán *et al.* ^59^
Increase in state subsidy revenues	Auxiliar variable	Pesos	0	Do not apply
Household income average	Stock	Pesos	1,250,000	Guzmán *et al.* ^59^
Impact of household income on entry age	Auxiliar variable	Years	GRAPH (Household_income_average) (0, 29.00), (500000, 29.00), (500001, 30.00), (1000000, 30.00), (1000001, 33.00), (1500000, 33.00), (1500001, 37.00), (2000000, 37.00), (2000001, 37.00), (2500000, 37.00), (2500001, 34.00), (3000000, 32.00), (3500001, 30.00), (4000002, 29.00), (4500003, 27.00), (5000004, 25.00), (5500005, 24.00), (6000006, 22.00), (6500007, 21.00)	Guzmán *et al.* ^59^
Income impact rate on obtaining credits and educational grants	Auxiliar variable	%	GRAPH (Household_income_average*Coverage_of_educational_credits_and_related_grants) (0, 0.012), (1000000, 0.012), (2000000, 0.012), (3000000, 0.012), (4000000, 0.012), (5000000, 0.012), (6000000, 0.014), (7000000, 0.014), (8000000, 0.014), (9000000, 0.014), (10000000, 0.014)	Guzmán *et al.* ^59^
Coverage of educational credits and related grants	Auxiliar variable	%	11	Ministry of National Education ^40^
Loans and educational support	Stock	Credits	1000	Ministry of National Education ^40^
Dropout rate due to lack of credits and educational grants	Auxiliar variable	%	GRAPH (Educational_credits_and_grants) (0, 0.153), (20000, 0.109), (40000, 0.077), (60000, 0.052), (80000, 0.024), (100000, 0.012), (120000, 0.000), (140000, 0.000), (160000, 0.000), (180000, 0.000), (200000, 0.000)	Guzmán *et al.* ^59^
P&GO Coverage	Auxiliar variable	%	8	Ministry of National Education ^40^
P&GO Interventions	Stock	Interventions	0	Do not apply
Drop-out rate due to ineffective P&GO interventions	Auxiliar variable	%	GRAPH(P&GO_Interventions) (0, 0.173), (1000000, 0.133), (2000000, 0.108), (3000000, 0.096), (4000000, 0.086), (5000000, 0.063), (6000000, 0.049), (7000000, 0.045), (8000000, 0.039), (9000000, 0.033), (10000000, 0.033)	Guzmán *et al.* ^59^

### Data

To parameterize the model, we used official sources of historical data from the Colombian State. The aggregate data of the level of higher education for the rural populations of the SPADIES, of the dropouts, as well as those of the Rural Higher Education Plan (PESR, according to its acronym in Spanish for Plan de Educación Superior Rural), ^25^ were taken. In the case of SPADIES, ^40^ the information can be freely consulted by registering on the application’s web portal; for the information extracted from the PESR, this was done through the link provided in Ref. 25. The data and sources are presented in Table 1.

Additionally, we used the database from the study by Guzmán *et al.,* ^59^ to develop some graphic functions (data and sources are in Table 1). A graphical function in system dynamics is a visual representation of how a variable changes over time in a dynamic model. The variable is represented on the vertical axis, while time is represented on the horizontal axis. These graphical functions can show how a variable varies as a function of initial conditions, system inputs and interactions between different variables in the model. From the above, graphical functions were developed by crossing two variables. An example of this was the average age of admission and the time people spend on their work duties, where from the scatter diagram generated, the points for the generation of the graphical function were taken.

### Simulations

With the model and the initial parameters (see Table 1), we proceeded to its execution and to evaluate public policies that could be implemented to mitigate and prevent the dropout in rural higher education in Colombia. For this, the following simulations were carried out (see Table 2). Additionally, Table 3 proposes the modification of the parameters in each of the simulations. In the case of SIM-2 to SIM-4, only one parameter was modified, so the other variables followed the *Ceteris Paribus* condition.

**Table 2.** Simulations.

Code	Description of simulation
SIM-1	It represents the simulation with the initial conditions of the system.
SIM-2	Public policy to increase the number of credits and educational support.
SIM-3	Public policy to additionally increase the average income of families.
SIM-4	Public policy for expansion of P&GO coverage by HEIs.
SIM-5	Política pública multidireccional con las condiciones dadas en SIM-2, SIM-3 y SIM-4

**Table 3.** Modification of initial parameters.

Code	Variable	Modification
SIM-1	Does not apply	Does not apply
SIM-2	Coverage of educational credits and related grants	30%
SIM-3	Increase in State subsidy revenues	$500.000 COP or USD 106.50
SIM-4	P&GO Coverage	35%
SIM-5	Does not apply	Modifications made in SIM-2, SIM-3 and SIM-4

Note: The exchange rate of COP to dollars taken as a reference was $4,694.71 COP per dollar.

For the present model, simulations were not conducted from a dynamic equilibrium due to three reasons. First, it is related to the DPM approach, which focuses on the adaptability and active improvement of the system’s performance in response to changes in the environment. This implies that the model can be analyzed and evaluated even if it is not in a state of dynamic equilibrium. Second, it is related to the objective of the article, which involves testing and evaluating how the system would react to different interventions and policies in a dynamic environment. In this case, a prior dynamic equilibrium is not necessarily required, as the focus is on exploring the dynamics and effects of policies on the system. Lastly, the third reason is related to data limitations. To establish a dynamic equilibrium, historical data for all variables in the system are required. However, the data obtained in the study by Guzmán *et al.* ^59^ were cross-sectional and not longitudinal, which hinders the establishment of a dynamic equilibrium.

### Analysis of simulation data

After conducting the simulations using the model and initial parameters from Table 1, the system’s behaviour was compared to SIM-1 using descriptive and inferential statistics. For SIM-1, the dominance of loops was evaluated using the analysis conducted by the Stella program. The goal was to determine the difference in means between the system’s behaviour with the initial parameters and the parameters modified by the simulations. Specifically, the Wilcoxon Sign Rank Test was used with a significance level of p-value < 0.05. ^60^ This analysis was limited to the levels of enrolled, graduated, and dropout rural students.

Finally, the computational work regarding the model and the simulations were implemented in the Stella Architect Software in version 3.3, ^61^ the model which is available in Ref. 62 can be replicated using Vensim. ^63^ The following model adjustments were taken into account: $t_{i}\;$ = 0, $t_{f}\;$ = 50, where $t_{i}\;$ represented the year 2016 first semester and $t_{f}$ the year 2041 semester two, likewise it was taken as Δt = 20, where t represents the time in academic periods; additionally, Euler was used as the integration method. The choice of the integration method was based on computational considerations and acceptability in approximating the simulation of public policy. Since the time period covered 25 years, an efficient execution method was required. While it is acknowledged that the Euler method can introduce a higher integration error, it provides a suitable approximation for our objective of obtaining a general understanding of the trends and behaviors of the system under study. In the case of statistical analysis, the SPSS Software version 28 was used. ^64^

## Results

In accordance with the methodological section, the results are presented in the four sections below. The first one shows the development of the DPM Chart; the second one, the Causal Loop Diagram; the third one, the validation of the model, and the fourth one presents the results of the simulations.

### DPM chart of dropout rates in rural higher education in Colombia

**Figure 1.**
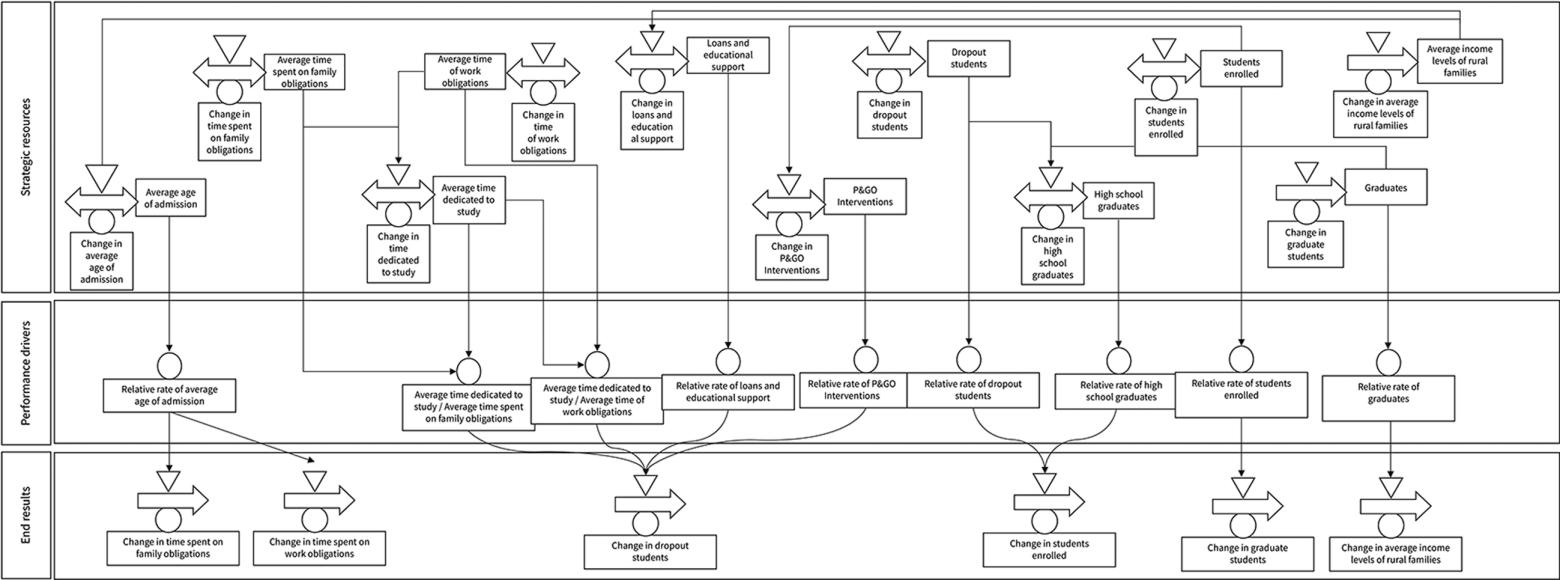
DPM chart of dropout in rural higher education in Colombia.

The DPM Chart table is a graphical representation tool that shows the interaction between strategic resources, performance drivers, and final outcomes of a system. To develop the DPM Chart, we have identified 11 strategic resources, nine performance drivers, and six end results (five outcomes and one output) as shown in Figure 1. The first strategic resource pertains to the average admission age to higher education, which means that the higher the age of admission, the more time students will spend on family obligations (second resource) and work (third resource), competing with the time devoted to study (fourth resource). ^2^^,^^32^

The average income levels of rural families (fifth resource) limit access to higher education, prompting the State to create and encourage programs to facilitate access to educational level. Low average income levels of families may lead to students being unable to enrol, resulting in them abandoning their educational process. ^5^ Western countries have provided loans and educational support (sixth resource) to rural students. HEIs have also created the P&GO (seventh resource) to intervene in variables of the individual determinant, especially those related to psychological aspects of the student, in addition to some of the academic and institutional determinants. In summary, the first and seventh resources impact the outcome of change in defectors.

The other four resources are: the number of dropouts (eighth), which will increase over time if the State and HEIs do not treat dropouts adequately; high school graduates (ninth), who are the potential population to enter higher education; enrolled students (tenth) corresponding to the population linked to a training program; and graduates (eleventh) who represent the population of students who obtained their degree. If students drop out, they will once again be part of the population of high school graduates.

In terms of the end results, we considered five outcomes, namely: the change in time spent on family obligations, the change in time spent on work obligations, the change in dropout students, the change in enrolled students, and the change in median family income. According to the logic of DPM, the outcomes are not entirely explained by the modelled structures, but other systems intervene for their full explanation. Regarding the outputs, we only identified one, which corresponds to the P&GO intervention.

### Causal loop diagram of dropout in rural higher education in Colombia

The causal loop diagram is a graphical representation of the causal relationships and feedback in a complex system. These diagrams allow for visualizing how variables in a system interact with each other and how these interactions generate positive or negative feedback effects. In this context, the reinforcing loop (R) is a structure in which changes in one variable generate adjustments that amplify those changes. The balancing loop (B) is a structure in which changes in one variable trigger adjustments that counteract those changes and tend to maintain the stability or equilibrium of the system.

**Figure 2.**
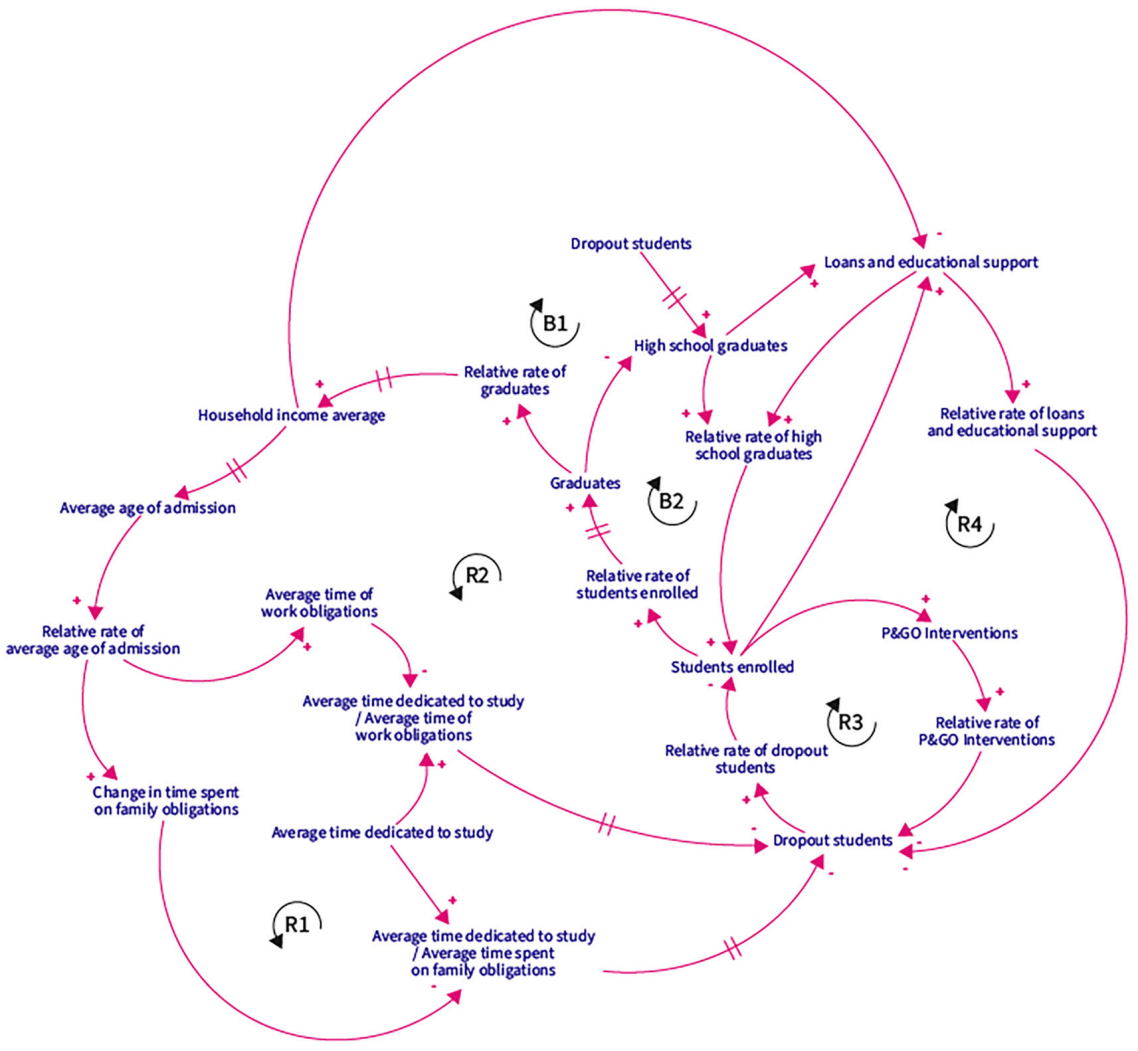
CLD. Note: R represents reinforcing loops and B balance loops. The sign “+” means proportional relationships, and “-” stands for inversely proportional relationships.

Based on the DPM Chart, and the interaction between strategic resources, performance drivers, and end results, a Causal Loop Diagram (CLD) was proposed with five reinforcement loops and two balancing ones. Figure 2 presents the CLD. The R1 loop shows that the higher the average age of admission to higher education, the more time students spend on family obligations, which decreases the time spent on studying, leading to more dropouts. The greater the number of dropouts, the lower the number of enrolees and graduates, making it impossible to increase the average family income, and thus, impact the average age of entry to higher education.

In the R2 reinforcement loop, if the average family income increases, students spend less time on family obligations since they have the financial support to delegate care responsibilities to third parties. This affects the time spent studying, decreases the number of dropouts, and increases the number of enrolees and graduates. As a result, more people with professional degrees in the family lead to a higher average family income.

In the R3 loop scenario, the higher the number of dropouts, the lower the number of students enrolled, which decreases the number of P&GO interventions, thus increasing the number of dropouts. Finally, the number of educational credits and supports for R4 depends on the number of high school graduates and enrolees. Thus, the greater the number of credits and aids, the lower the number of dropouts, more students enrolled, and more credits and educational aids granted.

Regarding the first balancing loop B1, the greater the number of dropouts, the fewer enrolees and graduates, decreasing the average family income. In the event of a low average family income, the number of credits and educational aid granted by the state will increase to prevent dropouts. For the B2 balancing loop, the greater the number of high school graduates, the higher the number of students enrolled in higher education, and the number of graduates, decreasing the number of people with only high school degrees.

Based on the CLD, a Forrester Diagram was developed for operationalizing the dynamic hypotheses. The diagram consisted of 11 stocks, 16 flows, and 22 auxiliary variables, which were used to define 32 equations, nine graphic functions, and six constants for the simulation model. These equations allowed for the development of the model and are presented in *Extended data.* ^68^ The simulation model is available in Ref. 62.

### Validation of the model of dropout in rural higher education in Colombia

**Figure 3.**
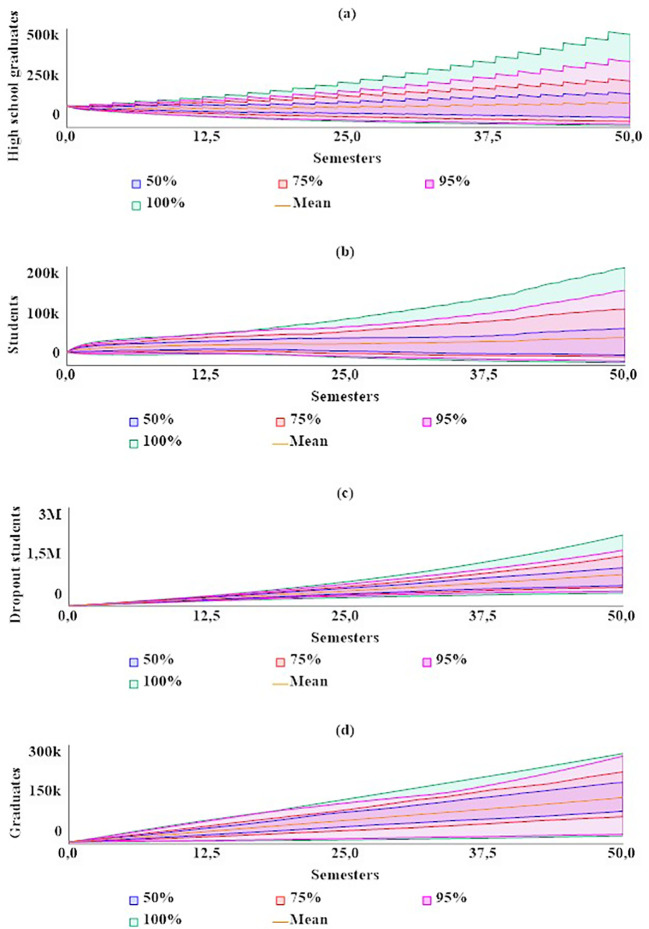
Sensitivity analysis for the dropout model in rural higher education in Colombia.

In the sensitivity analysis, the behavior of the system was evaluated for the stocks of high school graduates, students enrolled, dropout students, and graduates, as shown in Figure 3. Overall, numerical sensitivity was observed in all variables, indicating that the values of the stocks change significantly with variations in the parameters. However, despite these variations, a general pattern of stable behavior in the system was maintained.

**Figure 4.**
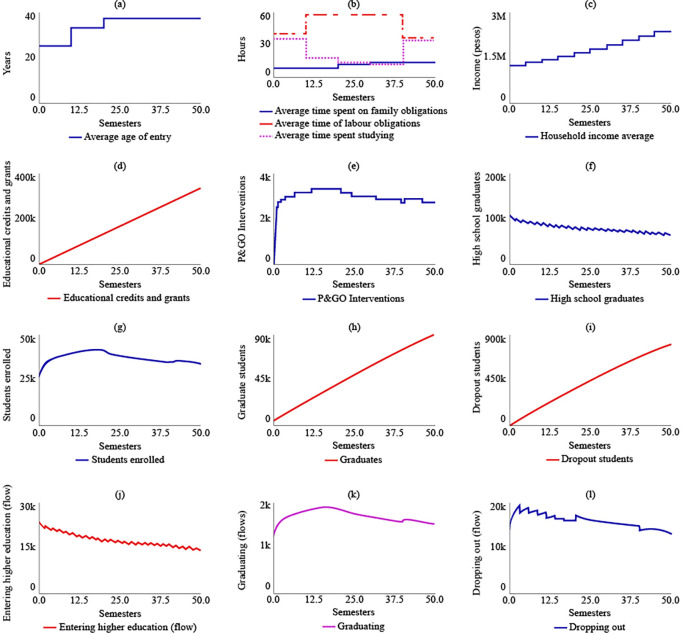
SIM-1 Results.

For the stock of high school graduates ( Figure 3a), it was estimated with a 95% confidence interval that, for t = 50, the value would range between 15,200 and 334,000. Within the same interval and period, for students enrolled ( Figure 3b), the value would range between 8,470 and 152,000. For dropout students ( Figure 3c), it would range between 457,000 and 1,710,000. Lastly, for graduates ( Figure 4c), the value would be between 29,300 and 267,000.

### Simulation results of dropout in rural higher education in Colombia

Under SIM-1, it was projected that the average age of admission to higher education for rural students in the year 2041 (t = 50) would be 37 years, an increase of 12 years from the year 2016 (t = 0). The simulation model predicted that rural students would spend 14 hours on family care, 35.5 hours on their jobs, and 34.5 hours on their studies for t = 50. The analysis of Figure 4b indicated that rural students would allocate only 13 hours to their training between t = 30 and t = 40, representing the shortest time for the simulated semesters.

The average family income would grow by 89.6% by 2041, reaching $2,380,000 COP (USD 506.95) due to the increase in the number of graduates in higher education, which was estimated to be 89,900 for t = 50. Educational loans and aid would impact 337,000 rural students if current policies are maintained. The number of P&GO interventions would be similar to the number of students enrolled, which was estimated to be 2.650 interventions for t = 50.

The simulation model predicted a decreasing trend in the number of students graduating from high school, which suggests that maintaining current policies will result in a greater link of students to higher education. For t = 50, it was estimated that 65,200 students will have a bachelor’s degree. The maximum number of students enrolled in higher education would be reached for t = 20, with 41,100 students, and the system’s behaviour would show a gradual decrease in enrolled students, ending at t = 50 with 33,800 students. The simulation model also predicted that the number of graduate students in the year 2041 would be 89,900, and the number of dropouts would be 808,000. The flow of enrolling would decrease, ending at t = 50 with 14,300 students starting their training, while the flows called graduating and dropping out would behave in a decreasing way in coherence with the decrease in enrolled students. Figure 4 displays the results of SIM-1.

**Figure 5.**
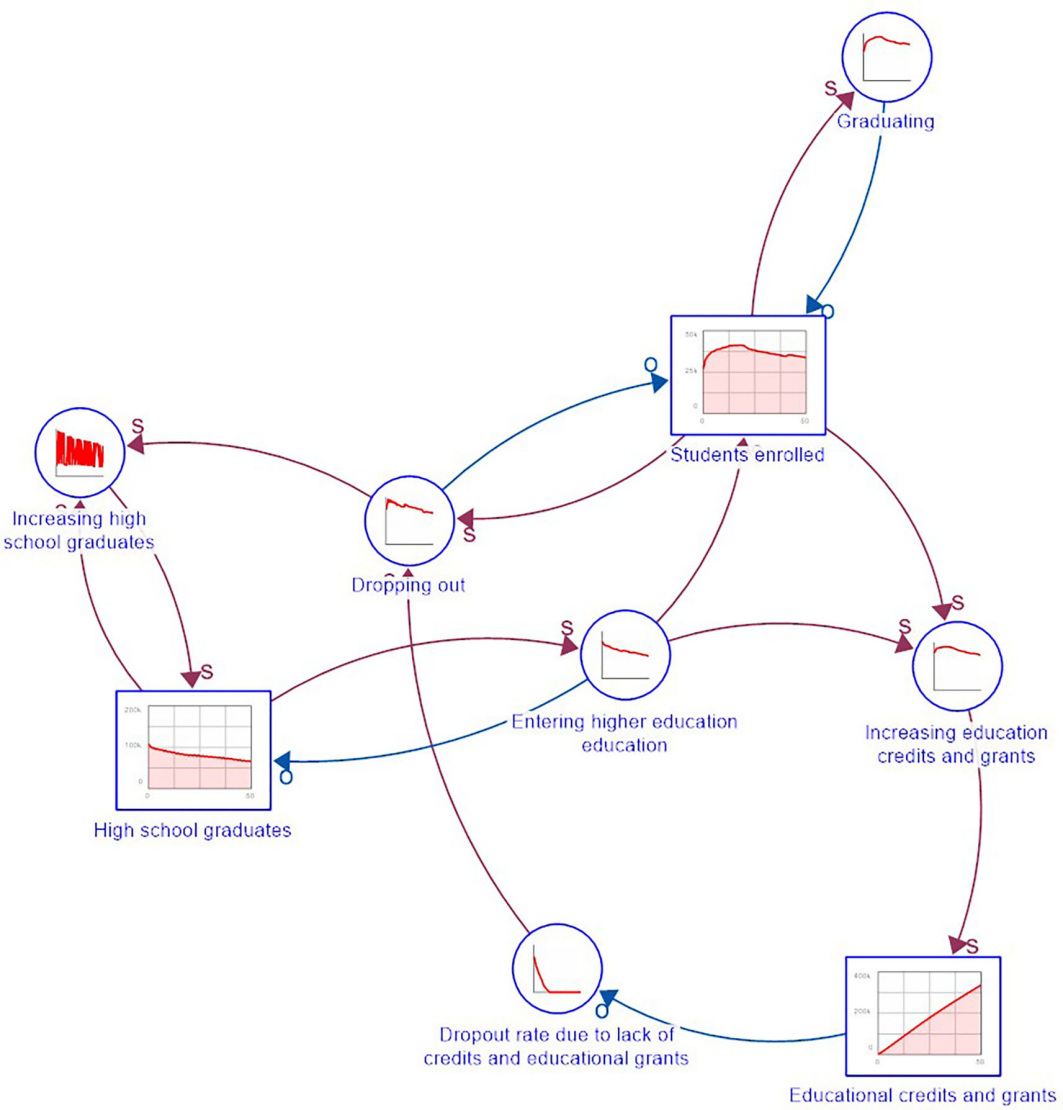
Dominance of causal loops in the model with initial parameters.

The system’s behavior is characterized by eight dominant causal loops (see Figure 5). The first balancing loop (Entering higher education → High school graduates → Entering higher education) explains 55.1% of the system’s behavior. The second balancing loop (Students enrolled → Dropping out → Students enrolled) accounts for 21.2% of the behavior. The third balancing loop (Students enrolled → Graduating → Students enrolled) represents 2.2% of the behavior. The fourth balancing loop (Increasing education credits and grants → Educational credits and grants → Dropout rate due to lack of credits and educational grants → Dropping out → Increasing high school graduates → High school graduates → Entering higher education → Increasing education credits and grants) contributes 0.41% to the system's behavior. The fifth balancing loop (Students enrolled → Increasing education credits and grants → Educational credits and grants → Dropout rate due to lack of credits and educational grants → Dropping out → Increasing high school graduates → High school graduates → Entering higher education → Students enrolled) accounts for 0.0041% of the behavior.

In terms of reinforcing loops, the first loop (Students enrolled → Dropping out → Increasing high school graduates → High school graduates → Entering higher education → Students enrolled) explains 20.8% of the system’s behavior. The second reinforcing loop (Students enrolled → Increasing education credits and grants → Educational credits and grants → Dropout rate due to lack of credits and educational grants → Dropping out → Students enrolled) represents 0.16% of the behavior. Finally, the third reinforcing loop (Increasing high school graduates → High school graduates → Increasing high school graduates) contributes 0.01% to the system’s behavior.

**Figure 6.**
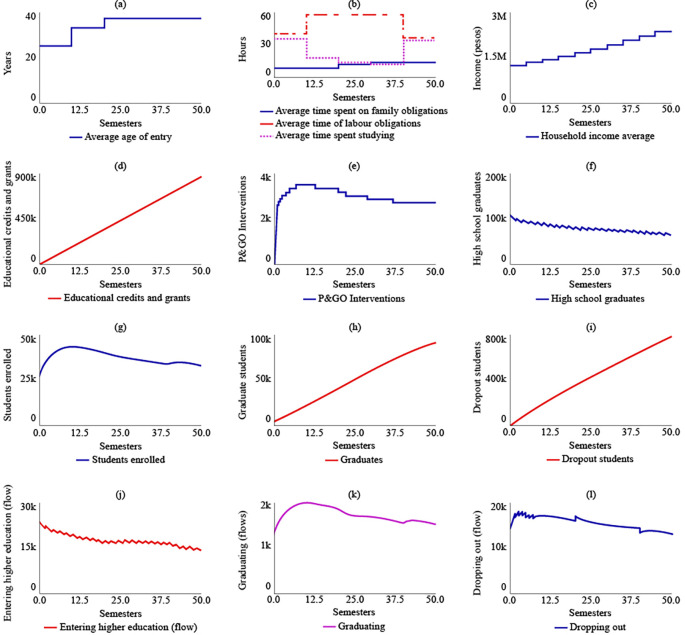
SIM-2 Results.

Under SIM-2, if the State were to allocate more educational credits and support to rural students, there would be no changes in the strategic resources compared to SIM-1. However, there would be a change in policy for t = 20, with the number of educational credits and supports increasing to 857,000. The maximum number of P&GO interventions would occur between t = 7 and t = 13, with 3,570 interventions. The system’s behaviour in terms of high school graduates, enrolled students, graduates, and dropouts would be similar to that of SIM-1. For t = 50, there would be 64,000 high school graduates, 33,100 enrolled, 90,600 graduates, and 786,000 dropouts. Figure 6 illustrates the behaviour of the system in the SIM-2 scenario.

**Figure 7.**
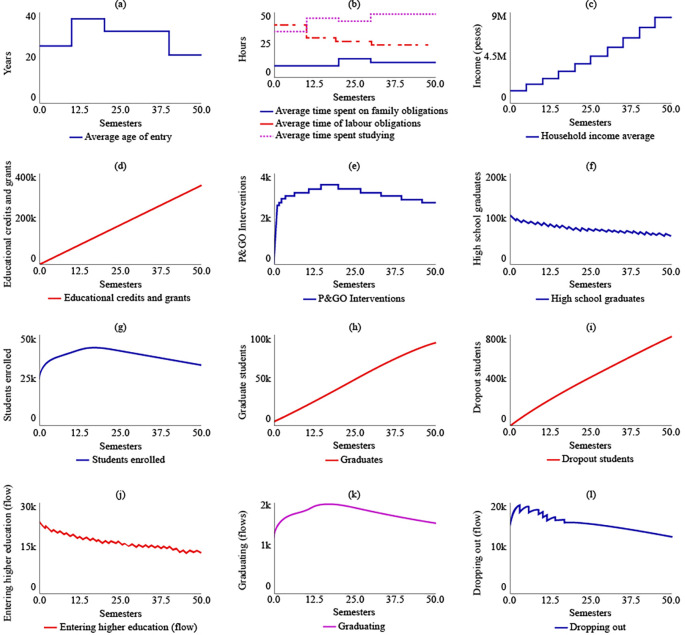
SIM-3 Results.

In the case of SIM-3, where there is an increase of $500,000 COP in the average family income, the average age of admission changes compared to SIM-1. Between t = 10 and t = 20, the average age increases by 12 years compared to the initial age of 25 years. However, after t = 20, the average age would decrease and reach 21 years for t = 50. As a result, there would be a readjustment in the distribution of the average time devoted to family, work, and study obligations, with 11.4, 24.7, and 48 hours allocated to each, respectively, for t = 50. In the year 2041, if the family income increase policy remains fixed due to the effect of State subsidies, the average family income is estimated to be $8,470,000 COP (USD 1,804.16). The number of educational credits and grants delivered would be $341,000 COP for t = 50. The maximum number of P&GO interventions would occur between t = 14 and t = 20, with 3,570 intervention sessions, and subsequently decrease. The behaviour of high school graduates would continue to decrease, with 61,000 for t = 50. However, the level variables of enrolled students, graduates, and dropouts would stabilize at 33,600, 92,600, and 773,000, respectively, for the year 2041. Figure 7 displays the results of SIM-3.

**Figure 8.**
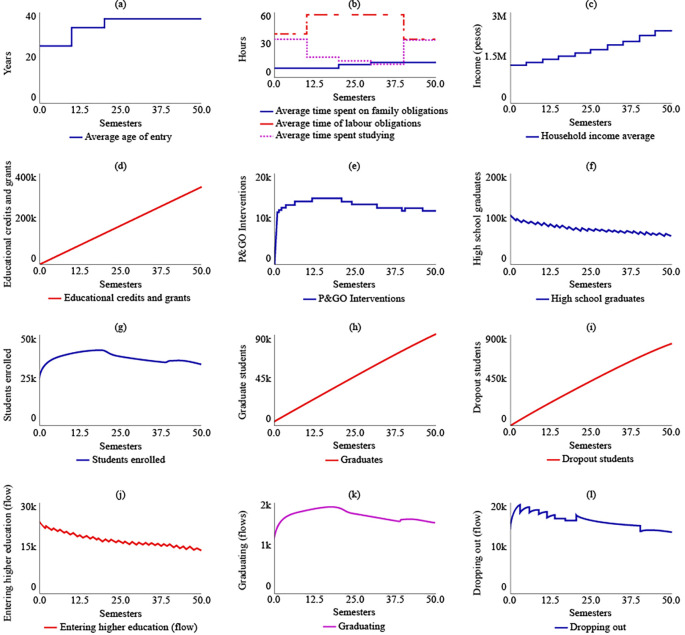
SIM-4 Results.

The results of the fourth simulation (SIM-4) indicate that the average age for entering higher education, as well as the average time spent on family, work, and study obligations, and the average family income, remain the same as those in SIM-1. In this scenario, it is expected that the State would have granted 337,000 educational loans and support. The maximum number of P&GO interventions would reach 14,900 students, and for t = 50, the interventions would be 11,600. The number of high school graduate students, enrolled students, graduates, and dropouts for t = 50 would be the same as those in SIM-1, which are 65,200, 33,800, 89,900, and 808,000, respectively. Figure 8 illustrates the system’s behaviour in SIM-4.

In SIM-5, a proposal for the simultaneous implementation of public policies, it is evident that this scenario would lead to a decrease in the average age of admission to higher education for rural populations, reaching 21.1 years by t = 50. As a result, there would be an increase in the average time dedicated to studying, which would be 48 hours by t = 50. In contrast, by 2041, the average time spent on work and family obligations would decrease by 24.7 and 11.4 hours, respectively. The average family income would increase due to State support and the income generated by the increase in the number of graduates. Thus, the family’s average income would be $8,470,000 COP (USD 1,804.16) by t = 50.

**Figure 9.**
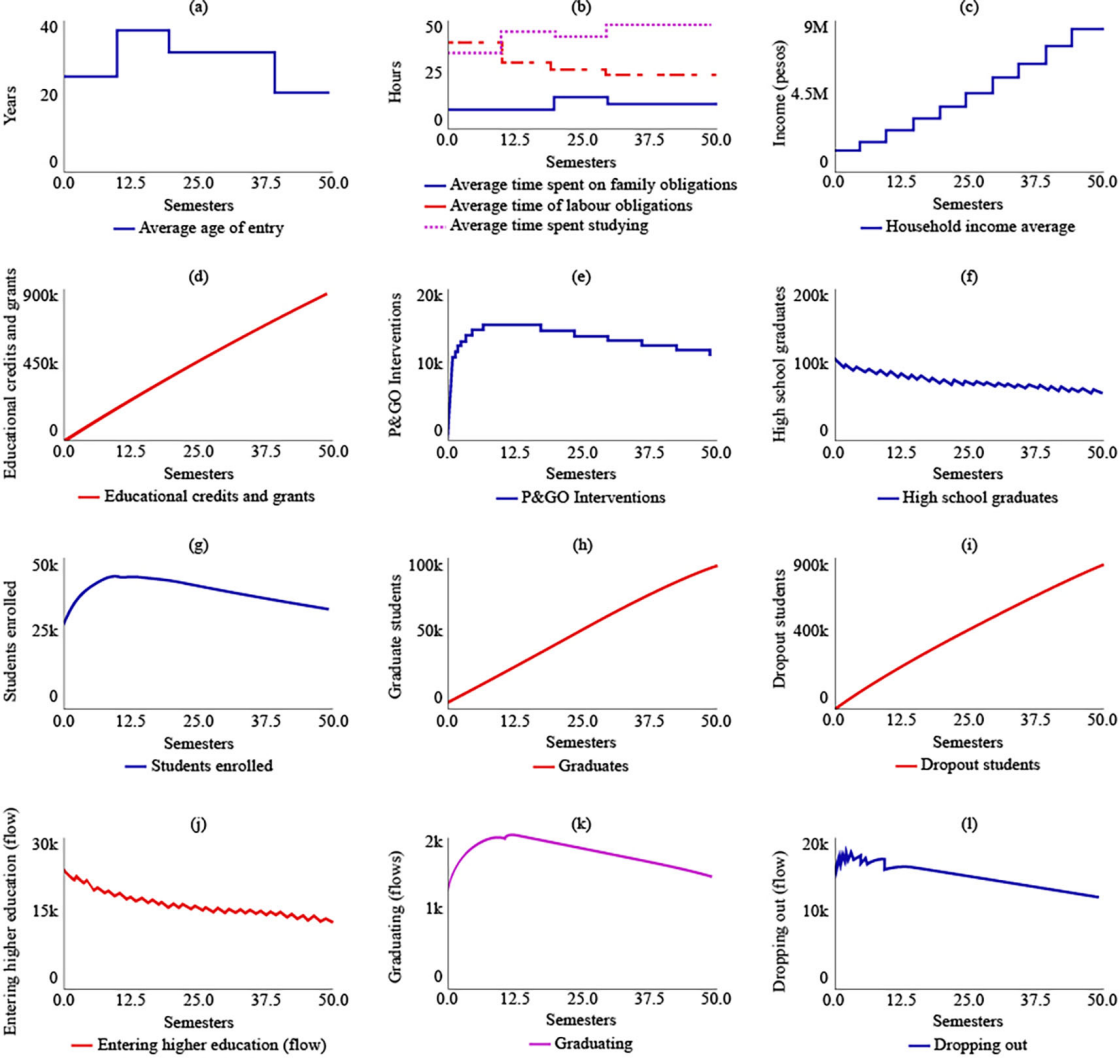
SIM-5 Results.

In this scenario of multiple policies, the number of subsidies delivered would reach 866,000 by t = 50. Similarly, between t = 6 and t = 18, it is estimated that the P&GO interventions would reach their maximum point at 15,600. After this period, the interventions would decrease, being equal to 11,600 by t = 50. As for the number of high school graduates by t = 50, there would be 59,900, with a decreasing curve from t = 1. The number of enrolees, graduates, and dropouts would be 32,900, 93,100, and 751,000, respectively, by t = 50. Figure 9 displays the behaviour of this simulation scenario.

From the results of the simulations, they were identified for the case of the number of students enrolled in higher education for SIM-2 (Z = -0.45, p-value = 0.65) and SIM-4 (Z = 0.60, p-value = 0.54). There would be no significant statistical differences with respect to SIM-1. Otherwise, in the scenario proposed in SIM-3, there would be significant differences with Z equal to -5.13 and a p-value less than 0.01, as well as in SIM 5 with Z equal to -5.47 and a p-value less than 0.01. With this in mind, for SIM-1, the average enrolment during the simulated periods was 37,182 students. For SIM-3, it was 38,231, and for SIM-5, it was 38,567. Regarding the number of dropouts, significant statistical differences were observed for SIM-2, SIM-3, and SIM-5 concerning SIM-1 (Z = -6.09, p-value < 0.01; Z = -6.17, p-value < 0.01; Z = -6.09, p-value < 0.01), only for SIM-4 it was not possible to determine the existence of said difference (Z = 0-90, p-value = 0.36). Thus, the mean number of student dropouts was 432,037 for SIM-1, 418,670 for SIM-2, and 404,870 for SIM-5. Finally, for the number of graduates, there were statistically significant differences in SIM-2, SIM-3, and SIM5 (Z = -6.09, p-value < 0.01; Z = -5.51, Z -value < 0.01; Z = -6.09, p-value < 0.01). The mean number of graduates was 48,689 for SIM-1, 49,681 for SIM-2, 49,910 for SIM-3, and 50,897 for SIM-5.

## Discussion and conclusions

The aim of this study was to simulate public policy scenarios for the treatment of school dropout in rural higher education in Colombia from a DPM approach. The model presented the strengthens of the literature on educational dropout in rural populations, by linking individual, socioeconomic, academic, and institutional determinants to the extent that it relates to dropout in higher education and rural populations over time. This includes variables such as age of entry to higher education, hours dedicated to studying, family and work obligations, educational credits, and financial support.

The simulation results demonstrate that expanding the coverage of educational loans and financial support for rural populations, which is the central axis of public policies in Western countries, ^65^ does not decrease the average age of admission to higher education. On the contrary, it increases it. This results in an increase in the time dedicated to family obligations ^32^ and work obligations, ^2^ which subsequently decreases the average time spent studying and increases the risk of dropout. However, this cannot be generalized for all simulated periods, as for t = 38, the time dedicated to studying increases while that dedicated to work obligations decreases. This is explained by the increase in average family income due to a greater number of people with professional degrees.

The implementation of policies that offer credits and educational aid for higher education in rural populations can indirectly decrease the probability of dropout due to payment and increase the number of graduates. The implementation of such a policy reduced the number of dropouts by 2.7% for t = 50. However, it should be considered whether indebtedness of the vulnerable rural population with limited economic income is the best strategy to prevent and mitigate dropout, as it can increase economic disparities that are already experienced in rural areas in developing countries. ^5^^,^^65^

An alternative public policy approach involves improving the average income of rural families, which can lower the age of admission to higher education for students from these areas. This eliminates the competition between the average times of labour obligations and relatives with those dedicated to studying. The implementation of this policy had an effect on the number of dropouts of 4.33%. This is possible since the average income of the family increases, benefiting all its members. This approach contrasts with Lewine *et al.,* ^23^ who indicated that subsidizing only the student would not reduce the number of dropouts because it generated pressure from the family to share their limited wealth, leading to partial or complete work and eventually dropout.

Regarding the increase in P&GO interventions resulting from changes in public policies regarding HEI quality criteria, there were no statistically significant differences for rural populations. This is due to the fact that the variables addressed in P&GO focus on academic, institutional, and individual (psychological) aspects that are difficult to modify through public policies, as stated by Guzmán *et al.,* ^66^ Castleman & Meyer, ^12^ Ramírez *et al.,* ^34^ and Lewine. ^23^

However, a simulation combining the three public policy options showed that their simultaneous application decreased dropout rates by up to 7.05%. This highlights the importance of coupling public policies beyond education to effectively and efficiently address the dynamic complexities of student dropout in higher education for rural populations, particularly in developing countries where social inequalities are pronounced.

While the developed model considered some of the most significant variables in studying dropout rates in rural populations, there are still opportunities for future research in various disciplines, such as the consideration of sex, prior academic competencies, academic and social capital at the start of higher education, and the type of IES, among others. It is also recognized that the incorporation of new variables may alter the effect of the evaluated public policies.

Although the data collected was already affected by COVID-19, it was not included as a study variable. However, it should be included in future model replications if the data does not already include this specific situation. Additionally, as there is a lack of related data in Colombia regarding the dropout rate from the educational system, the model assumes a continuity of the flow of students. However, once data on this variable is available, it must be incorporated, thus affecting the absolute amount of each level variable but not the system’s behaviour.

## Data Availability

DANS-EASY: Simulation Results, https://doi.org/10.17026/dans-zjb-5rsd. ^67^ This project contains the following underlying data:
• Dataset of simulations.csv Figshare: Extended data, https://doi.org/10.6084/m9.figshare.22658989.v2. ^68^ This project contains the following extended data:
•
Equations used by the model.docx Data are available under the terms of the Creative Commons Attribution 4.0 International license (CC-BY 4.0).
