# Supplementary material for: Prevention and Mitigation of Rural Higher Education Dropout in Colombia: A Dynamic Performance Management Approach
Source: F1000Res. 2023 Jun 1;12:497. Originally published 2023 May 15. [Version 2] doi: 10.12688/f1000research.132267.2 (PMC10329195; doi:10.12688/f1000research.132267.2)
Supplement: Peer review history

# Peer review: Reviewer response for version 2

**DOI:** 10.5256/f1000research.148547.r181512

**Published:** 2023-07-07

**Authors:** Lamprini Trivella

**Affiliations:** 1 University of West Attica, Athens, Greece

This article addresses an issue that concerns the universities globally. It is appropriate theme and it is easily conceivable. It adds important elements in the research about the hindrance and decrease of dropouts in the rural higher education in Colombia by using a dynamic performance management approach. This is very important in Colombia according to the high-rate percentages of rural higher education dropouts. I have approved the manuscript for publication. At this point, this paper is highly readable, the tables and the figures were readable too.

 Notably, the manuscript is clear and transparent, citing the current literature (for example: Guzmán A, Barrag el of simulation. Isee Exchange. 2023.; Bivona E: Determinants of performance drivers in online food delivery platforms: a dynamic performance management perspective. IJPPM. 2022. etc).

 The methods used are sufficiently explained and the interpretation of data is precise ( for example: pg.6). All the important data are described in a usable and accessible format (for example: pg.7, 8, 9). The conclusions are deducted according to the results, highlighting the limitations of this research and making suggestions for further research (for example: pg. 19-20).

Is the work clearly and accurately presented and does it cite the current literature?

Yes

If applicable, is the statistical analysis and its interpretation appropriate?

I cannot comment. A qualified statistician is required.

Are all the source data underlying the results available to ensure full reproducibility?

Yes

Is the study design appropriate and is the work technically sound?

Yes

Are the conclusions drawn adequately supported by the results?

Yes

Are sufficient details of methods and analysis provided to allow replication by others?

Yes

Reviewer Expertise:

Distance Higher Education, Comparative education, Cultural Organizations’ Management, 3D computer animation production

I confirm that I have read this submission and believe that I have an appropriate level of expertise to confirm that it is of an acceptable scientific standard.

# Peer review: Reviewer response for version 2

**DOI:** 10.5256/f1000research.148547.r176268

**Published:** 2023-06-05

**Authors:** Robinson Salazar Rua

**Affiliations:** 1 Center for Community Health Integration, Case Western Reserve University, Cleveland, Ohio, USA

The authors have properly addressed all my concerns and I believe the quality of the manuscript has improved significantly.

Is the work clearly and accurately presented and does it cite the current literature?

Yes

If applicable, is the statistical analysis and its interpretation appropriate?

Yes

Are all the source data underlying the results available to ensure full reproducibility?

Yes

Is the study design appropriate and is the work technically sound?

Partly

Are the conclusions drawn adequately supported by the results?

Yes

Are sufficient details of methods and analysis provided to allow replication by others?

Yes

Reviewer Expertise:

NA

I confirm that I have read this submission and believe that I have an appropriate level of expertise to confirm that it is of an acceptable scientific standard.

# Peer review: Reviewer response for version 1

**DOI:** 10.5256/f1000research.145177.r173754

**Published:** 2023-05-18

**Authors:** Robinson Salazar Rua

**Affiliations:** 1 Center for Community Health Integration, Case Western Reserve University, Cleveland, Ohio, USA

The paper is highly readable, informative, and transparent. It furthers research on the treatment of dropout in rural higher education by using a Dynamic Performance Management (DPM) Approach. This is an important topic in Colombia, and I have approved the manuscript with reservations. At this point, improvements are needed but they could be addressed by taking into account the following suggestions.

 1) In the design section, I suggest expanding the discussions of the DPM approach since it is the main method to reach the objective of the article. In particular, I recommend deepening on the concepts of strategic resources, performance drivers, and end/results. In addition, it is pertinent to clarify what the added value of the DPM is in respect to the Causal Loop Diagram (CLD) and Forrester Diagram.

 2) In the last lines of the design section, it was mentioned that the consistency of the model was tested by comparing simulation results with the suggested behavior from the causal loop diagram (i.e., reference mode). I will encourage the authors to use other tests to increase the reliability and robustness of the model. Below you will find two references to support such endeavor. 

- Barlas Y (1996) Formal aspects of model validity and validation in system dynamics. Syst Dyn Rev 12(3):183–210
- Schwaninger, M., Groesser, S. (2020). System Dynamics Modeling: Validation for Quality Assurance. In: Dangerfield, B. (eds) System Dynamics. Encyclopedia of Complexity and Systems Science Series. Springer, New York, NY. https://doi.org/10.1007/978-1-4939-8790-0_540

Moreover, the authors might consider conducting sensitivity analyses to determine the robustness of the model and its associated policies under parameter uncertainty. In addition, they might analyze loop dominance by using the new feature in Stella Architect that was incorporated for such purpose. This supports the identification of important leverage points in the system by means of an analytical approach. 

 3) In the first lines of the simulation section, it says “With the model and the initial parameters, we proceeded to its execution and to evaluate public policies that could be implemented to mitigate and prevent the dropout in rural higher education in Colombia”. After reading this line, one question came to my mind. Was the model set in dynamic equilibrium before testing the policies? It is considered as a best practice to do it. However, it is not always the case. I think it would be beneficial to the reader, who has a background in system dynamics, to clarify this question. 

 4) In the analysis of the simulation data, perhaps the time step is 2 instead of 20. However, I would like the authors to confirm it. I would assume the time step to be 2 since the model is running in a period of 25 years (from 2016 to 2041), the initial time is zero and the final time is 50 semesters. 

 In case the authors confirm the time step to be 20, I would like to know why the Euler, instead of Runge Kutta 4th order, method was selected. The higher the time step, the higher the integration error. When the time step is high, it’s better to use more efficient integration methods to reduce the calculation errors by the software. 

 5) In the results section, I would suggest putting the Table 1 (Data and sources of model parameterization) in an appendix. In addition, I would suggest making bigger Figure 1. After printing the article, I noticed the names of the strategic resources, performance drivers, and end-results are too small and it’s difficult to read. In addition, I would recommend renaming Figure 1 by using a more illustrative name for the case study than just naming it as DPM chart. 

 6) In the section Causal loop and Forrester Diagrams, the authors immediately start discussing the reinforcing and balancing loops from the CLD. However, I think it would be beneficial to spend some lines on explaining what a balancing and reinforcing loop is for those readers who are not familiarized with such concepts. In addition, I was doubting whether it would be beneficial to rename the section since the Forrester Diagram was cited in the last paragraph but, unlike the CLD, it was not explicitly displayed in the article by means of a figure. 

 7) It would be beneficial to sort the references in alphabetical order. 

 8) I also suggest the following book and article to complement the relevant ideas - related to the education sector in the Colombian context- t hat were discussed throughout this article. 

- Salazar Rua R. 2023. Improving consistency in performance measurement system design: The case of the Colombian public schools. Switzerland: Springer Cham. 
- Bianchi C, Salazar Rua R. 2020. A feedback view of behavioral distortions from perceived public service gaps at ‘street-level’ policy implementation: The case of unintended outcomes in public schools. System Research and Behavioral Science. pp: 1–22. 

Is the work clearly and accurately presented and does it cite the current literature?

Yes

If applicable, is the statistical analysis and its interpretation appropriate?

Yes

Are all the source data underlying the results available to ensure full reproducibility?

Yes

Is the study design appropriate and is the work technically sound?

Partly

Are the conclusions drawn adequately supported by the results?

Yes

Are sufficient details of methods and analysis provided to allow replication by others?

Yes

Reviewer Expertise:

System Dynamics, Performance Management, Performance Measurement, Dynamic Performance Management, Systems Science, Computational modeling, Education policies, Health policies

I confirm that I have read this submission and believe that I have an appropriate level of expertise to confirm that it is of an acceptable scientific standard, however I have significant reservations, as outlined above.

## References

[1] : Formal aspects of model validity and validation in system dynamics. *System Dynamics Review* .1996;12(3) : 10.1002/(SICI)1099-1727(199623)12:3<183::AID-SDR103>3.0.CO;2-4 183-210 10.1002/(SICI)1099-1727(199623)12:3<183::AID-SDR103>3.0.CO;2-4

[2] : Renal surface reconstruction and segmentation for image-guided surgical navigation of laparoscopic partial nephrectomy. *Biomed Eng Lett* .2023;13(2) : 10.1007/s13534-023-00263-1 165-174 10.1007/s13534-023-00263-1 37124114PMC10130295

[3] : Improving consistency in performance measurement system design: The case of the Colombian public schools. *Switzerland: Springer Cham.* .2023;

[4] : A feedback view of behavioural distortions from perceived public service gaps at ‘street‐level’ policy implementation: The case of unintended outcomes in public schools. *Systems Research and Behavioral Science* .2022;39(1) : 10.1002/sres.2771 63-84 10.1002/sres.2771

# Response

**Published:** 2023-05-20

**Authors:** Alfredo Guzmán Rincón

**Affiliations:** Corporación Universitaria de Asturias, Colombia

Dear PhD. Robinson Salazar Rua,

 We sincerely appreciate the time you dedicated to reviewing our work. Likewise, we believe that each of your comments has helped strengthen our article, especially regarding the simulation model. With that said, we will proceed to address each of the points and how they were addressed in the revised version of the article.

 Comment 1: In the design section, I suggest expanding the discussions of the DPM approach since it is the main method to reach the objective of the article. In particular, I recommend deepening on the concepts of strategic resources, performance drivers, and end/results. In addition, it is pertinent to clarify what the added value of the DPM is in respect to the Causal Loop Diagram (CLD) and Forrester Diagram.

 Response 1: In the initial version of the article, we hadn't considered clarifying aspects related to the DPM approach. Therefore, in the methods section, under the design subsection, we added a description of the DPM approach and its differentiation from the Causal Loop Diagram (CLD) and Forrester Diagram. Additionally, we provided definitions for the concepts of strategic resources, performance drivers, and end/results.

 Comment 2: In the last lines of the design section, it was mentioned that the consistency of the model was tested by comparing simulation results with the suggested behavior from the causal loop diagram (i.e., reference mode). I will encourage the authors to use other tests to increase the reliability and robustness of the model. Below you will find two references to support such endeavor. • Barlas Y (1996) Formal aspects of model validity and validation in system dynamics. Syst Dyn Rev 12(3):183–210 • Schwaninger, M., Groesser, S. (2020). System Dynamics Modeling: Validation for Quality Assurance. In: Dangerfield, B. (eds) System Dynamics. Encyclopedia of Complexity and Systems Science Series. Springer, New York, NY. https://doi.org/10.1007/978-1-4939-8790-0_540

 Moreover, the authors might consider conducting sensitivity analyses to determine the robustness of the model and its associated policies under parameter uncertainty. In addition, they might analyze loop dominance by using the new feature in Stella Architect that was incorporated for such purpose. This supports the identification of important leverage points in the system by means of an analytical approach.

 Response 2: Considering the suggestion regarding model validation, we reviewed the recommended references and made adjustments to the validation process. In this regard, the selected method was sensitivity analysis based on the consistency of the model's behavior. This change led to an expansion of the results section. Additionally, in the simulation results section for SIM-1, we added the dominance of causal loops generated by the system.

 Comment 3:      In the first lines of the simulation section, it says “With the model and the initial parameters, we proceeded to its execution and to evaluate public policies that could be implemented to mitigate and prevent the dropout in rural higher education in Colombia”. After reading this line, one question came to my mind. Was the model set in dynamic equilibrium before testing the policies? It is considered as a best practice to do it. However, it is not always the case. I think it would be beneficial to the reader, who has a background in system dynamics, to clarify this question. 

 Response 3: In response to this comment, the simulation section was amended to include the following text: "For the present model, simulations were not conducted from a dynamic equilibrium due to three reasons. First, it is related to the DPM approach, which focuses on the adaptation and active improvement of the system's performance in response to changes in the environment. This implies that the model can be analyzed and evaluated even if it is not in a state of dynamic equilibrium. Second, considering the objective of the article, it involves testing and evaluating how the system would react to different interventions and policies in a dynamic environment. In this case, there is no strict requirement for a prior dynamic equilibrium since the focus is on exploring the dynamics and effects of policies in the system. Lastly, the limitation of data availability plays a role since establishing dynamic equilibrium requires historical data for all variables in the system, and the data obtained in the study by Guzmán et al. [55] were cross-sectional rather than longitudinal."

 Comment 4:  In the analysis of the simulation data, perhaps the time step is 2 instead of 20. However, I would like the authors to confirm it. I would assume the time step to be 2 since the model is running in a period of 25 years (from 2016 to 2041), the initial time is zero and the final time is 50 semesters. In case the authors confirm the time step to be 20, I would like to know why the Euler, instead of Runge Kutta 4th order, method was selected. The higher the time step, the higher the integration error. When the time step is high, it’s better to use more efficient integration methods to reduce the calculation errors by the software. 

 Response 4: Regarding the comment, indeed, the method used was Euler with a Δt = 20. The decision to use Euler as the integration method was based on computational considerations and acceptability in the approximation of the public policy simulation. In this regard, the following considerations were taken into account:

1. Euler's method is computationally more efficient than the 4th order Runge-Kutta method, especially when using a larger time step. Since the simulation data analysis is conducted over a period of 25 years with a time step of 20, using Euler's method allows for faster and more efficient execution of the model.
2. Although Euler's method may introduce a higher integration error compared to the 4th order Runge-Kutta method, in certain cases, it can provide an acceptable approximation of the results. Given the overall objective of the article, which is to understand the effect of implementing public policies rather than focusing solely on precision, Euler's method was deemed suitable.

These reasons were explained to the readers of the article in the section on data analysis methods.

 Comment 5: In the results section, I would suggest putting the Table 1 (Data and sources of model parameterization) in an appendix. In addition, I would suggest making bigger Figure 1. After printing the article, I noticed the names of the strategic resources, performance drivers, and end-results are too small and it’s difficult to read. In addition, I would recommend renaming Figure 1 by using a more illustrative name for the case study than just naming it as DPM chart. 

 Response 5: The journal does not handle supplementary materials or appendices, which is why the table is included in the main text. Regarding Figure 1, a request has been made to the Editor to allocate a full page for it in the new version. Additionally, the name of the Figure has been adjusted.

 Comment 6: In the section Causal loop and Forrester Diagrams, the authors immediately start discussing the reinforcing and balancing loops from the CLD. However, I think it would be beneficial to spend some lines on explaining what a balancing and reinforcing loop is for those readers who are not familiarized with such concepts. In addition, I was doubting whether it would be beneficial to rename the section since the Forrester Diagram was cited in the last paragraph but, unlike the CLD, it was not explicitly displayed in the article by means of a figure.

 Response 6: A conceptual introduction was provided in the section where the Causal Loop Diagram (CLD) is presented. Additionally, the section name has been adjusted to accurately reflect its content. Considering the valuable contribution, the same adjustments have been made to the DPM section.

 Comment 7: It would be beneficial to sort the references in alphabetical order.

 Response 7: According to the journal's guidelines, references must be numbered and cannot be organized alphabetically.

 Comment 8: I also suggest the following book and article to complement the relevant ideas - related to the education sector in the Colombian context that were discussed throughout this article. 

- Salazar Rua R. 2023. Improving consistency in performance measurement system design: The case of the Colombian public schools. Switzerland: Springer Cham. 
- Bianchi C, Salazar Rua R. 2020. A feedback view of behavioral distortions from perceived public service gaps at ‘street-level’ policy implementation: The case of unintended outcomes in public schools. System Research and Behavioral Science. pp: 1–22. 

Response 8: The suggested documents were reviewed, and some of the ideas in the article were complemented with them. Additionally, other documents were also consulted and incorporated to enhance the content.
